# Hydrofilm Polyurethane Films Reduce Radiation Dermatitis Severity in Hypofractionated Whole-Breast Irradiation: An Objective, Intra-Patient Randomized Dual-Center Assessment †

**DOI:** 10.3390/polym11122112

**Published:** 2019-12-16

**Authors:** Leonard Christopher Schmeel, David Koch, Frederic Carsten Schmeel, Bettina Bücheler, Christina Leitzen, Birgit Mahlmann, Dorothea Kunze, Martina Heimann, Dilini Brüser, Alina-Valik Abramian, Felix Schoroth, Thomas Müdder, Fred Röhner, Stephan Garbe, Brigitta Gertrud Baumert, Hans Heinz Schild, Timo Martin Wilhelm-Buchstab

**Affiliations:** 1 Department of Radiology and Radiation Oncology, University Hospital Bonn, Venusberg-Campus 1, 53127 Bonn, Germany; 2 Radiotherapy Bonn-Rhein-Sieg, Practice at academic St. Marien Hospital, Robert-Koch-Str. 1, 53115 Bonn, Germany; 3 Department of Gynecology and Obstetrics, Division of Senology, University Hospital Bonn, University of Bonn, Venusberg Campus 1, 53127 Bonn, Germany; 4 Institute of Radiation Oncology, Graubuenden Cantonal Hospital, Loestr. 170, 7000 Chur, Switzerland; 5 Radiotherapy Bonn-Rhein-Sieg, Practice at academic Protestant Johanniter Clinics Bonn, Waldstr. 73, 53177 Bonn, Germany

**Keywords:** radiation dermatitis, erythema, breast cancer, Hydrofilm, film dressing, whole-breast irradiation, hypofractionation

## Abstract

Radiation-induced skin injury represents the most frequent side effect in breast cancer patients undergoing whole-breast irradiation (WBI). Numerous clinical studies on systemic and topical treatments for radiation dermatitis have failed to provide sustainable treatment strategies. While protective skin products such as dressings are undoubtedly the standard of care in wound care management, their utilization as preventive treatment in radiotherapy has been somewhat neglected in recent years. In this prospective, intra-patient randomized observational study, Hydrofilm polyurethane films were prophylactically applied to either the medial or lateral breast-half of 74 patients with breast cancer undergoing hypofractionated whole-breast irradiation following breast-preserving surgery. Maximum radiation dermatitis severity was assessed using Common Terminology Criteria for Adverse Events (CTCAE) v4.03 toxicity scores, photospectrometric erythema and pigmentation measurements and patient-assessed modified Radiation-Induced Skin Reaction Assessment Scale (RISRAS) scale. Phantom studies revealed a clinically negligible dose build-up of less than 0.1% with Hydrofilm. Compared to the control compartments physician-assessed radiation dermatitis severity was reduced in the hydrofilm compartments (mean 0.54 vs. 1.34; *p* = < 0.001). Objective photospectrometric skin measurements showed decreased erythema (*p* = 0.0001) and hyperpigmentation (*p* = 0.002) underneath Hydrofilm. Hydrofilm also completely prevented moist desquamation, and significantly reduced patients’ treatment-related symptoms of itching, burning, pain, and limitations of day-to-day-activities. Significant beneficial effects were observed in terms of radiation dermatitis severity, erythema, hyperpigmentation as well as subjective treatment-related symptom experiences, while adverse reactions were rare and minor. Therefore, a prophylactic application of Hydrofilm polyurethane films can be suggested in hypofractionated WBI.

## 1. Background

Whole-breast irradiation (WBI) represents the standard of care after breast-conserving surgery for breast cancer as it significantly improves oncologic outcomes following lumpectomy [1].

Conventional fractionation regimens in WBI are typically performed over a period of 5–6 weeks with fractions (fx) of 1.8–2 Gy per day. Randomized trials have shown equivalent rates of overall survival and local control with hypofractionated WBI, which uses slightly higher doses per fx, delivered over a shorter treatment period (usually 2.5–3.33 Gy/fx) [2]. This more rapid completion of therapy also reduces overall health care expenditures, so hypofractionated WBI has become the preferred adjuvant treatment in WBI [3,4,5,6,7].

Even though there is rising evidence that hypofractionated WBI results in slightly lower acute skin toxicity, the most frequent acute complication of hypofractionated WBI still remains the radiation dermatitis with swelling, erythema, desquamation, burning, and pain of the irradiated integument [8,9]. These reactions occur during the course of radiotherapy and can last for weeks post treatment [10]. Radiation dermatitis is not only among the patients’ best-known side effects associated with WBI, but has also been shown to significantly affect their quality of life [8].

Numerous clinical trials therefore evaluated the prevention of acute radiation dermatitis. These applied topical treatments covered a vast variety from steroidal to non-steroidal treatments. However, the majority of trials reported only little efficacy and were not able to provide efficacious and sustainable treatment or prevention strategies [11,12,13].

A relatively novel approach for treatment and prophylaxis of radiation-induced skin reactions is based on skin protection products in the form of polymeric barrier-forming solutions or superficial skin-covering layers such as dressings or films. The underlying concept assumes that skin damage may be prevented by maintenance of the keratinized surface and protection of the radiotherapy-affected basal stem-cell layer from superficial abrasion and friction [14,15,16,17,18,19]. Barrier-forming products also reduce transepidermal water loss secondary to radiation and can thereby ameliorate radiation dermatitis [20].

Despite the fact that initial promising results have been obtained with barrier-forming products, Lim et al. and Wan et al. recently outlined that this approach has barely attracted attention in radiotherapy [21,22].

We recently investigated the prophylactic application of polyurethane films (Hydrofilm) in an initial feasibility study and observed a reduction of both incidence and severity of radiation dermatitis during normofractionated WBI [23].

Hydrofilm is a transparent and quite thin polyurethrane film (150 µm) sticking to the skin surface by a hypoallergenic acrylic adhesive and is approved as a primary dressing to cover post-operative and trauma wounds or as a secondary dressing for retention purposes [24]. The films are sterile, semi-permeable, water proof as well as bacteria proof, and can remain on the skin for several weeks without causing trauma upon removal.

Since hypofractionation has been established as the preferred treatment schedule in WBI for early breast cancer, we sought to extend the few experiences in the field of polymeric barrier-forming skin products for the reduction of radiation-induced skin reactions with patients receiving exclusively hypofractionated WBI, particularly as hypofractionation is supposed to be associated with less radiation-induced skin reactions [8,9]. The objective was to evaluate whether and to what extent a prophylactic Hydrofilm application benefits patients receiving hypofractionated WBI.

## 2. Materials and Methods

### 2.1. Participants

From March 2018 until June 2019, patients receiving WBI at our comprehensive academic cancer center and at a participating community radiation oncology practice were screened for recruitment.

Inclusion criteria were: age > 18 years, breast-preserving surgery for breast cancer and a fractionation regimen of 40.05 Gy in 15 fractions (fx).

To avoid a potential bias of the obtained radiation dermatitis gradings as much as possible we defined the following exclusion criteria: neoadjuvant/concomitant chemotherapy, active smoking status, metastatic disease, previous radiation to the ipsilateral breast, breast reconstruction, active dermatitis, any preexisting dermatological disorders, treatment with topical or oral corticosteroids, mastectomy, different fractionation regimens, tattoos in the irradiation area, and patient refusal to participate.

### 2.2. Study Methodology

The irradiated breast of each patient was divided into medial and lateral halves randomized by computer-generated randomization protocols to either Hydrofilm (Paul Hartmann AG, Heidenheim, Germany) or urea lotion (Eucerin UreaRepair PLUS Lotion 5% Urea, Beiersdorf AG, Hamburg, Germany) as control skin care.

Preceding the first WBI, Hydrofilm was applied on either the lateral or medial breast compartment by a radiation oncologist and replaced upon detachment. Care was taken to achieve a smooth and unwrinkled attachment to avoid creases or patient discomfort.

Patients were given oral and written information to apply the urea lotion twice a day to the irradiated control compartment, starting at the first treatment day. Patients were encouraged not to use any complementary topical treatment. Compliance was checked with at least one weekly visit. Patients presenting with radiation dermatitis grade ≥ 2 with moist desquamation and intense pain were prescribed topical corticosteroids, if necessary.

### 2.3. Radiation Protocol

Patients received a fractionation regimen of 40.05 Gy in 15 fractions using 6 MV or a combination of 6 and 10 MV photons following 3D planning, either with tangential photon beams as sliding-window intensity modulated radiotherapy (IMRT) or volumetric arc therapy (VMAT). For invasive cancer cases, a sequential normofractionated tumor-bed boost of 16 Gy with 2 Gy/fx was given to patients with a positive or close-margin following surgical resection, patients age 50 and younger, and patients age 51 to 70 if they had a high-grade tumor.

Patients were treated on a Varian TrueBeam STx (Palo Alto, California, USA) linear accelerator in a supine position. Left-sided WBI was performed in deep-inspiration breath-hold. International Commission on Radiation Units and Measurements (ICRU) recommendations for dose limits of 95% to 107% were followed.

### 2.4. Patient Evaluation

Patients were evaluated at the weekly standard on-treatment visit; any additional visits requested by the patients were also recorded. Radiation dermatitis assessments were reported according to the National Cancer Institute Common Terminology Criteria for Adverse Events (CTCAE) v4.03 [25]: Grade 0 = no dermatitis; grade 1 = faint erythema or dry desquamation; grade 2 = moderate to brisk erythema, patchy moist desquamation, mostly confined to skin folds and creases, moderate edema; grade 3 = moist desquamation in areas other than skin folds and creases, bleeding induced by minor trauma or abrasion; grade 4 = life-threatening consequences, skin necrosis or ulceration of full thickness dermis, spontaneous bleeding from involved site, skin graft indicated; grade 5 = death.

Upon completion of WBI, Hydrofilm dressings were removed and the patient-assessed modified Radiation-Induced Skin Reaction Assessment Scale (RISRAS) was recorded [26]. Patients reported their maximum experience of itching, burning, pain, and limitations in daily activities. For each of these items 0–3 points were given by the patient (0 = not at all, 1 = a little, 2 = quite a bit, 3 = very much).

**Figure 1:**
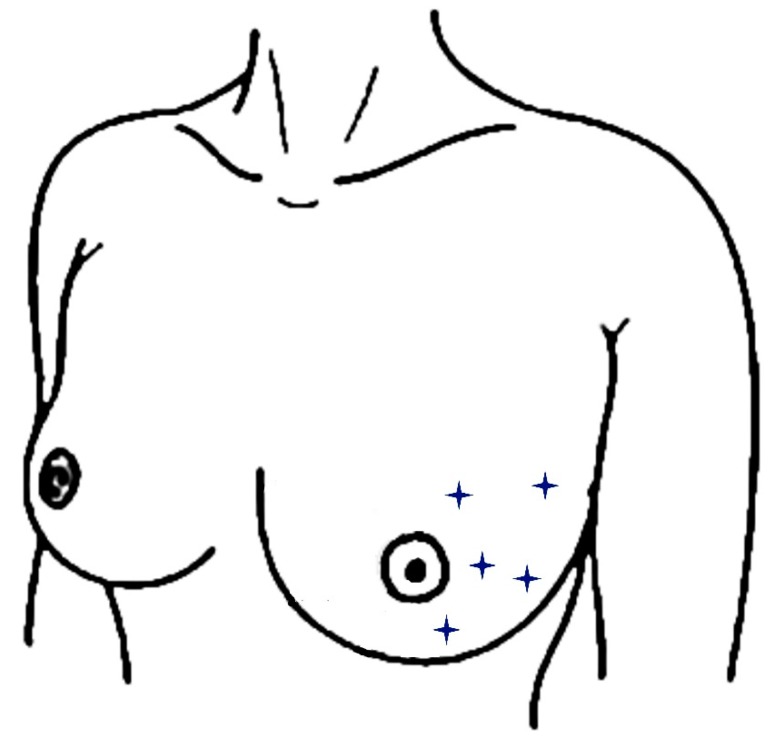
Locations of spectrophotometric skin readings within a breast half. Measurement locations are indicated as stars and were performed analogously within the other breast half.

The day after completion of WBI, five erythema readings across the Hydrofilm-covered as well as the control area (see Figure 1) were performed with a reflectance spectrophotometer (CR-10 Plus, Konica-Minolta, Marunouchi, Japan) [16,23,27].

This compact device is applied on the patient’s skin without touching it, and measurements are performed automatically. The measurements are based on the CIE (Commission Internationale de l’Eclairage) system of tristimulus values describing each measured color by three coordinates using the L*a*b* coordinate system; the L* value describes the luminance, the a* value describes the position of the color on a scale ranging from red to green, and the b* value the position on a scale ranging from blue to yellow (of subordinate importance in our study). Accordingly, higher L* values describe lighter skin and higher a* values are interpreted as increased erythema intensity [25]. According to Momm and Russell, a* values between 0 and 10 describe unirradiated skin or faint erythema, values of 10–15 desribe mild erythema, and values up to 25 describe very intense erythema [16,23,28,29]. In addition, the skin condition was documented by digital photography (Canon EOS 1300 D, Canon Inc., Tokyo, Japan).

### 2.5. Statistical Analysis

Mean, median, range, and standard deviations (SD) were calculated for all applicable clinical data, unless otherwise stated. The two-tailed Wilcoxon signed-rank test was used for pairwise comparisons of ordinal-scaled dermatitis gradings and patient-assessed RISRAS scores among both breast compartments, either with or without hydrofilm. After ensuring homogeneity of variances with the Levene-test, interval-scaled spectrophotometric measurement data were compared via the one-sample, two-tailed *t*-test. Odds ratios (OR) and 95% confidence intervals (95% CI) were calculated for physician-assessed data. Post-hoc statistical power was evaluated for mean differences of physician-assessed data. Box-and-whisker plots were generated to indicate the degree of dispersion and skewness in the data, and show outliers. The statistical significance level was defined as p ≤ 0.05. SPSS Statistics v25 (IBM, Armonk, New York, USA) was used for data analysis.

### 2.6. Phantom Studies

To determine possible dose variations caused by Hydrofilm, percentage depth dose (PDD) measurements were performed with a RW3 Slab phantom [Physikalisch-Technische Werkstätten (PTW), Freiburg, Germany] at depths of 7, 9, 11, 13, 15, 17, 20, 25, 30, and 50 mm. PDDs were measured with an Advanced Markus ion chamber (PTW) with or without Hydrofilm on the surface with a source-to-surface distance of 100 cm. Three measurements were taken for each depth with the Unidos webline (PTW) electrometer for both 6 and 10 MV photon beams.

## 3. Results

We enrolled 80 patients between March 2018 and June 2019. 74 patients completed the study as intended and yielded data for analysis. Patient characteristics are shown in Table 1. Three patients withdrew consent, three patients stopped applying Hydrofilm within the first five days due to itching sensations (n = 2), redness of the skin (n = 3), and allergic eczema (n = 2), including coincidence.

### 3.1. Phantom Studies

Phantom studies revealed insignificantly increased doses of less than 0.1% with Hydrofilm. The resulting dose build-up was therefore considered as clinically negligible and Hydrofilm remained on the skin over the entire WBI period.

### 3.2. Effect on Severity of Radiation Dermatitis and Desquamation as Assessed by CTCAE v4.03 Scores

**Figure 2:**
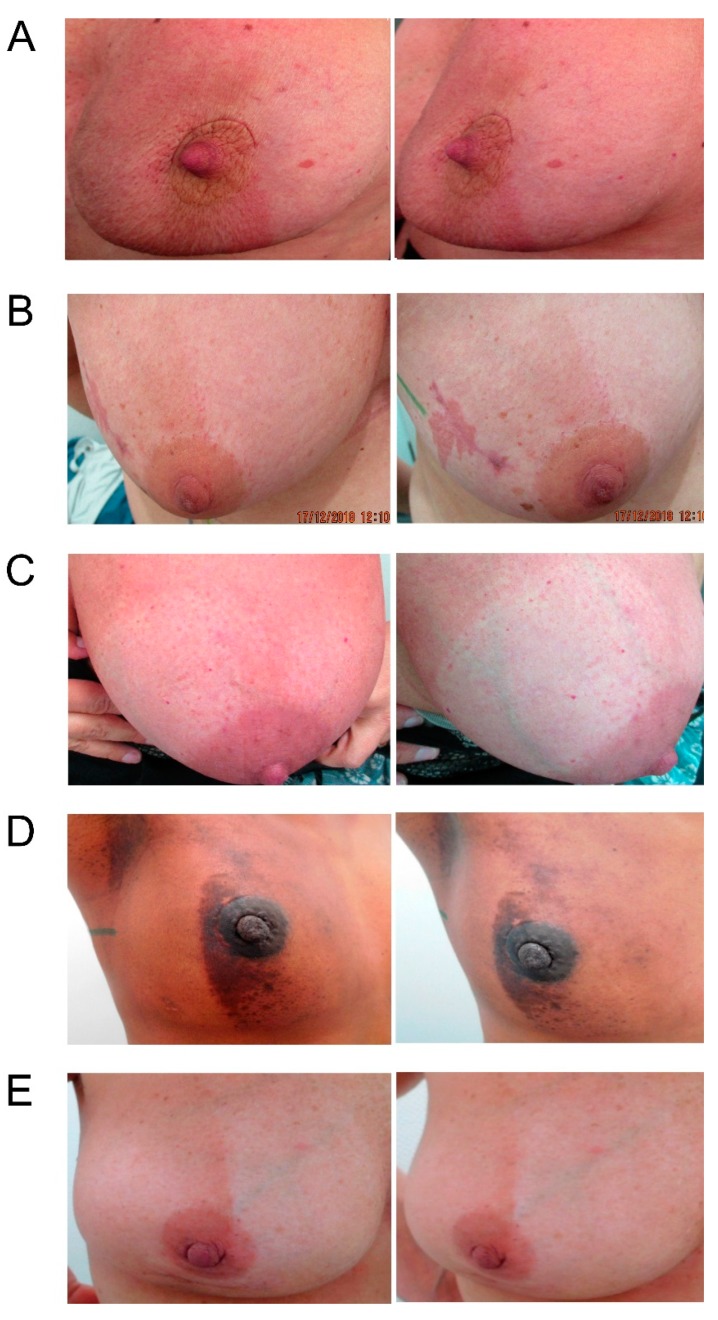
Exemplary photographs, each taken from two different angles, of five (**A**–**E**) different patients following completion of the whole-breast irradiation. Hydrofilm was applied to the lateral breast compartment in patients **A** + **D** and to the medial breast compartment in patients **B**, **C**, and **E** during the entire therapy period.

Maximum severity of radiation dermatitis was significantly lower within the Hydrofilm-covered breast. Mean scores were 0.54 ± 0.56 (95% CI 0.41–0.67) under Hydrofilm, and 1.34 ± 0.63 (95% CI 1.19–1.49) in the control compartment, respectively. Mean difference was 0.8 (*p* = < 0.001); post-hoc evaluation of mean differences showed a statistical power of 100% (α =0.05 and β = 0.2). Dermatitis severity grades ≥ II occurred in 9.5% vs. 36.5% of patients (*p*= < 0.001; OR= 10.07 (95%CI: 3.86-26.28)). Five patients (6.8%) presented with moist desquamation in the control areas, while none was seen under the film. Dry desquamation occurred in 2 (2.7%) vs. 25 (33.8%) patients with and without Hydrofilm (*p* = < 0.001; OR= 18.89 (4.26–83.7)). Five patients (6.8%) required topical corticosteroids for the treatment of their control compartments due to intense burning sensations or pain. The results are summarized in Table 2 and exemplary photographs of patients on completion of the WBI are shown in Figure 2.

### 3.3. Objective Assessment of Maximum Erythema Severity and Pigmentation by Reflectance Spectrophotometry

**Figure 3:**
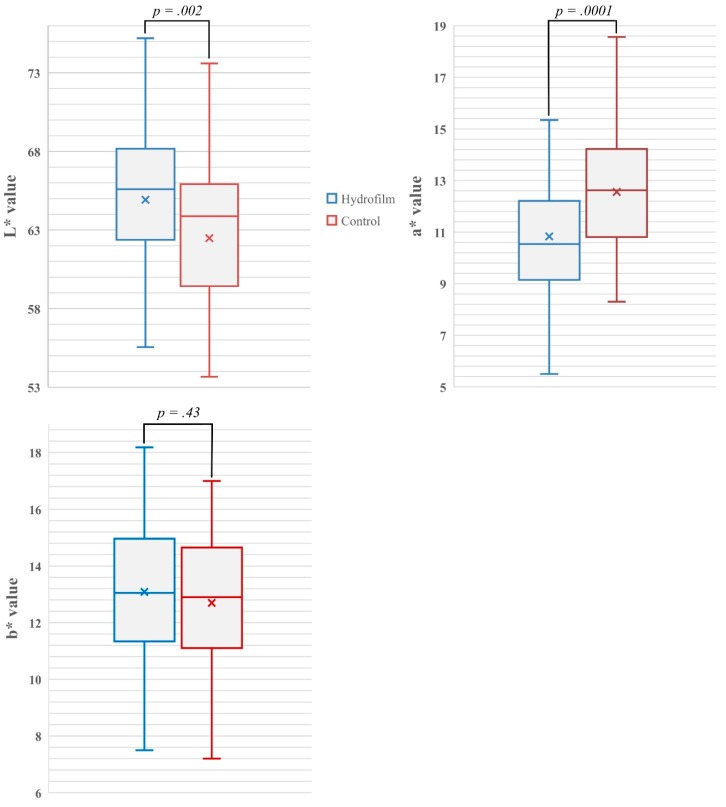
Box- and whisker-plots showing the mean (indicated as X) and median (indicated as band inside) differences, and the range of measured L*-values as indicator of skin pigmentation, a*-values as indicator of erythema severity and b* values in both the Hydrofilm-covered and control breast compartments. Increased L*-values indicate increased skin luminance/less pigmentation; increased a*-values indicate increased erythema; b* values describe the color position on a scale ranging from blue to yellow.

Erythema severity was significantly lower under Hydrofilm: mean and median a* values were 10.83 ± 2.48 (95% CI 10.57–11.08) and 10.54 with Hydrofilm, and 13.16 ± 2.61 (95% CI 12.89–13.42) and 12.74 in the control compartments, respectively (*p* = 0.0001). Hydrofilm also prevented skin hyperpigmentation with mean and median L* values of 65.31 ± 4.31 (95% CI 64.87–65.74) and 65.6, compared with 59.9 ± 4.87 (95% CI 59.40–60.39) and 60.86 without Hydrofilm, respectively (*p* = 0.002). No significant differences were observed in b* values; mean and median b* values were 13.08 ± 2.49 (95% CI 12.51–13.65) and 13.05 with Hydrofilm and 12.7 ± 2.51 (95% CI 12.13–13.27) and 12.9 without Hydrofilm, respectively (*p* = 0.43). See differences in pigmentation and erythema severity in Figure 3.

### 3.4. Subjective Experience of Itching, Burning, Pain and Limitations in Daily Activities

Patient-assessed modified RISRAS scores showed that patients were significantly less bothered by treatment-related itching, pain, burning, and limited day-to-day activities following Hydrofilm application; mean scores were 0.22, 0.18, 0.12, and 0.07 compared to 0.95, 0.72, 0.74, and 0.24 in the control compartments, respectively. Corresponding *p*-values were < 0.001 for itching, pain and burning, and 0.006 for limitations in day-to-day activities. Of patients, 57 (77%) preferred Hydrofilm to cream and were convinced that their symptoms could be reduced. We found that 66 (89%) patients would recommend Hydrofilm to patients during WBI.

### 3.5. Adverse Reactions Induced by Hydrofilm Dressings

Adverse reactions induced by Hydrofilm were minor and can be distinguished in reactions underneath the film, and those solely occurring at the boundaries of the film resulting from local shear stress. Two patients developed an allergic response immediately and two other patients developed a delayed reaction with maculopapular rash following the first Hydrofilm application.

Mild itching sensations and skin redness were seen in six (8.1%) and eight (10.8%) patients, but only present at the very edge of the film. The latter two symptoms occurred due to shear stress from inaccurate Hydrofilm placement which was, unfortunately, not apparent to the treating radiation oncologist.

All observed adverse reactions (see Table 3) were mild and self-limiting without additional therapy.

### 3.6. Cost-Benefit Consideration

Costs of Hydrofilm dressings were approximately 10 € per patient, whereas the costs of concurrent prophylactic treatment in the control breast-halves were approximately 20 € per patient. Even more important, patients requested both more frequent patient visits and radiation oncologists’ time due to more severe skin injury in the non-film covered areas (on average two additional visits). Five patients also required topical corticosteroids for the treatment of their control compartments (approximately 25 € per patient).

## 4. Discussion

Hypofractionated WBI is meanwhile preferred to normofractionated WBI as it is associated with less chronic reactions and improved long-term cosmesis [4]. While there is growing evidence of decreased acute radiation dermatitis following hypofractionated WBI, skin injury still remains the most frequent acute side effect [3,5]. Particularly the proximity to clothing, frequent movement of the arms, and perspiration cause a continuous mechanical and physical irritation of the breast. These factors render the irradiated skin susceptible to radiation-induced skin reactions. Although protection from friction and maceration by dressings is the standard of care in damaged skin areas (e.g., burns), this approach has not been widely appreciated in radiation-induced skin injury [21,23,30].

The underlying concept assumes that superficial skin protection may reduce damage to the skin surface and the subjacent basal-stem layer by absorbing physical and chemical impacts caused by friction, perspiration, or maceration. Such a cover-protected skin environment also maintains or even increases the skin hydration required for an accelerated reepithelialization [17,20,31,32].

So far, seven studies investigated varying barrier-forming skin products in terms of a preventive application during photon radiotherapy. Two of these evaluated Cavilon in patients receiving postmastectomy irradiation. Cavilon is a polymeric solution forming a film when applied to the skin and usually applied as a barrier against irritation from body fluids. In the first series, the frequency of moist desquamation was reduced from 46% to 33% and dermatitis severity was also reduced. However, a subsequent study found neither a statistical difference for skin reactions nor for moist desquamation rates between treatment and control arms [15,16]. However, another randomized study with head and neck squamous cell cancer (HNSCC) patients investigated a quite similar, film-forming silicone fluid and reported significantly reduced dermatitis rates [33].

Three studies evaluated the prophylactic application of silicone dressings (Mepitel Film, Mölnlycke Healthcare, Gothenburg, Sweden) compared with aqueous cream during radiotherapy of HNSCC and breast cancer patients. Skin reaction severity and frequency of moist desquamation was decreased by at least 27% and 28%, respectively, in HNSCC patients [32]. In 78 breast cancer patients, moist desquamation rates were reduced from 26% to 0%, and skin reactions were significantly reduced in those areas covered prophylactically with silicone-dressings [17]: peak skin reactions were grade 0, I, II, and III in 0%, 28%, 64%, and 8%, respectively, in the control areas while 56%, 36%, and 8% of patients developed grade 0, I, and II skin reactions after prophylactic placement of silicone-patches using a comparable study design with an intra-patient randomization. The prophylactic efficacy of Mepitel film was recently reinvestigated in WBI and post-mastectomy radiotherapy [34]. A significant reduction of radiation dermatitis severity was reported in patients undergoing post-mastectomy radiotherapy and in patients treated with a total dose of 50 Gy during WBI.

Patients also reported a significant lower level of pain, itching, burning sensation as well as edema and reduced sensitivity, irrespective of the dose-fractionation regimen used. Although no significant physician-assessed dermatitis differences were found in those patients treated with hypofraction (*p*= 0.1), grade ≥ II dermatitis occurred less frequently with 7% vs. 14% following prophylactic Mepitel film placement vs. standard skin care. While these silicone-dressings are products with different material characteristics, the basic principle of superficial skin protection is similar. Differences between Hydrofilm and Mepitel film are not only the differing materials, but also that Hydrofilm adheres more firmly to the skin surface due to the acrylic adhesive; a resulting advantage is that they are less easily detached, e.g., during showering or exercise. A potential disadvantage might be that premature removal could be more difficult and also unpleasant for the patient. In our initial feasibility experience with Hydrofilm in patients undergoing normofractionated WBI, we observed a significantly decreased dermatitis severity under Hydrofilm with grade 0 radiation dermatitis in 48.2% vs. 12.5% and grade ≥ 2 in 12.5% vs. 41.1% of patients following either a prophylactic Hydrofilm application or standard care, respectively [23].

Dermatitis severities of our patients’ control skin compartments were comparable to the recent literature [8,9,34] while the polyurethane-film-covered integument presented with significantly reduced dermatitis rates with a considerable mean reduction of peak CTCAE score by 0.8, and significantly reduced erythema and hyperpigmentation in reflectance spectrophotometry. In particular, the higher dermatitis severity levels (≥ grade 2) could be reduced to approximately a third of patients (9.6% vs. 36.5%) and dermatitis could be completely prevented in approximately three times as many patients compared to standard care (44.6% vs. 14.9%). These results are in line with the data obtained in our initial experience with Hydrofilm in normofractionated WBI [23] and show a comparable efficacy in reducing radiation dermatitis severity. Importantly, treatment-related symptoms such as itching, pain, burning, and limited day-to-day activities were also significantly reduced in skin areas covered by Hydrofilm.

The current series is the first to specifically address the efficacy of a prophylactically applied barrier-forming skin product in patients undergoing exclusively hypofractionated WBI, the current WBI treatment standard in early breast cancer.

We included spectrophotometric measurements to allow for an objective skin color assessment and to augment the reliability of our physician-evaluated observations as previously performed in several studies assessing radiation dermatitis [23,27,28,29,35]. These measurements facilitate reliable, reproducible color records and also ensure the detection of subtle differences which may not be noticed during visual skin inspections [35,36]. To our knowledge, such technical measurements have not yet been applied by any other workgroup in the field of barrier-forming skin protection products, although pure subjective dermatitis assessments were shown to be compromised by considerable intra-evaluator variations and inter-evaluator differences [36,37].

Owing to its negligible bolus effect and high wearing comfort, Hydrofilm remained on the skin during daily treatments without any limitations of the radiation therapy and patient activities such as showering, workout, or wardrobe selection. Average costs of Hydrofilm were lower than for our standard skin care and additional costs for topical treatments, and more frequent patient visits were saved.

However, optimal application of the Hydrofilm requires some attention to details. In our previous feasibility study, we observed some side effects at the periphery of the Hydrofilm dressings, especially skin redness (19.4%), mild itching (16.1%) and burning sensations (11.3%). These reactions occurred due to shear stress at the edges following a suboptimal film application (e.g., in the mammary or axillary fold). Steadily improved placement skills based on the experiences gained in our former series may explain the greater tolerability and declined rate of side effects. Contrary to our feasibility study, adverse reactions were comparably rare but also low in severity and self-limiting without any additional therapy.

A highly uniform treatment site and waiving different fractionation schedules are important advantages of the current study. The intra-patient randomization design excludes patient-related aspects possibly influencing our findings. The use of validated scoring instruments, patient-reported symptom differences and especially the use of objective technical measurements render our data robust. However, we acknowledge some limitations: due to the visibility of Hydrofilm on the skin-surface, blinding of both patients and radiation oncologists was not feasible. In a few previous series, increased dermatitis rates were observed 1–3 weeks following hypofractionated WBI [38]. Another limitation is, therefore, that no skin measurements/scorings were performed after therapy completion. However, a potentially delayed dermatitis peak was not merely disregarded; the rationale behind this was to ensure comparability of the obtained data since 25 of 74 patients received a sequential local boost radiotherapy to the former tumor bed.

## 5. Conclusions

Our series underpins the presumed principle of prophylactic superficial skin protection for the reduction or even the prevention of radiation-induced skin toxicity. We demonstrate a considerable reduction of radiation dermatitis following a prophylactic Hydrofilm application with a very reasonable cost-benefit ratio. Significant beneficial effects were also observed in terms of erythema, hyperpigmentation, and desquamation, as well as subjective symptom experiences, while adverse reactions were minor and rare. Therefore, a prophylactic application of Hydrofilm polyurethane films can be recommended in WBI.

## 6. Disclosure Statement

### Ethics Approval and Consent to Participate

This observational dual-center, intra-patient randomized clinical study was approved by the local Ethics Committee (186/2016), and written informed consent was obtained by all participants. The study was conducted in accordance with the Declaration of Helsinki.

## Figures and Tables

**Table 1:** Patient baseline characteristics.

Characteristic	Total
Total enrolled	80
Total completed	74
Randomization: Hydrofilm medial	37
Randomization: Hydrofilm lateral	37
Gender	
Female	79
Male	1
Age	
Median age	62
Mean Age	60.31
Age range	37–84
Ethnicity	
Caucasian	75
Turkic	4
Pardo Brazilian	1
T Classification (TNM)	
DCIS	17
T1	45
T2	18
Radiation therapy	
40.05 Gy in 15 fractions	74
Sequential boost (16 Gy in 8 fx)	25 *
Mean dose to PTV in % of prescribed dose	
Medial compartment	103
Lateral compartment	103
V_≥107%_ in cm³ ± SD	
Medial compartment	33.5 ± 65
Lateral compartment	34.5 ± 50

Abbreviations: TNM= TNM Classification of Malignant Tumors; DCIS= ductal carcinoma in situ; fx = fraction; PTV = planning target volume (whole breast); V_≥107%_ = PTV receiving ≥107% of prescribed dose; SD = standard deviation; * Radiation dermatitis gradings, modified Radiation-Induced Skin Reaction Assessment Scale (RISRAS) scoring and spectrophotometric skin measurements were performed following the application of 40.05 Gy in 15 fractions in all patients to ensure data comparability as a sequential boost dose is likely to increase skin toxicity.

**Table 2:** Maximum severity of radiation dermatitis corresponding to the CTCAE v4.03 score in 74 patients.

CTCAE Score	Hydrofilm Compartment	Control Compartment	P	Odds Ratio (95% CI)
	Number of Patients (%)	Number of Patients (%)		
0	33 (44.6)	11 (14.9)		
I	34 (45.9)	36 (48.6)		
II	7 (9.5)	27 (36.5)		
III	0	0		
Mild dermatitis grade ≤ I	67 (90.5)	47 (63.5)	<0.001	5.5 (2.2–13.7)
Moderate dermatitis grade ≥ II	7 (9.5)	27 (36.5)	<0.001	10.1 (3.9–26.3)
Dry desquamation	2 (2.7)	25 (33.78)	<0.001	18.9 (4.3–83.7)
Moist desquamation	0	5 (6.8)	0.09	11.8 (0.6–217.2)
Patients requiring topical corticosteroids	0	5 (6.8)	0.09	11.8 (0.6–217.2)
Abbreviations: CTCAE = Common Terminology Criteria of Adverse Events; CI = Confidence Interval				

**Table 3:** Adverse reactions caused by Hydrofilm dressings.

Adverse Reaction	Number of Patients	Percentage (%)
Localized maculopapular rash *	4	5.2
Mild itching ^†^	6	8.1
Mild skin redness ^†^	8	10.8

* Including three patients who withdrew prior to study completion; ^†^ Adverse reactions were restricted solely to the peripheral area of Hydrofilm dressings.
